# Probable rapid eye movement sleep behavior disorder, nocturnal disturbances and quality of life in patients with Parkinson’s disease: a case-controlled study using the rapid eye movement sleep behavior disorder screening questionnaire

**DOI:** 10.1186/1471-2377-13-18

**Published:** 2013-02-09

**Authors:** Keisuke Suzuki, Tomoyuki Miyamoto, Masayuki Miyamoto, Yuka Watanabe, Shiho Suzuki, Muneto Tatsumoto, Masaoki Iwanami, Tsubasa Sada, Taro Kadowaki, Ayaka Numao, Kenichi Hashimoto, Hideki Sakuta, Koichi Hirata

**Affiliations:** 1 Department of Neurology, Dokkyo Medical University, 880 Kitakobayashi, Mibu, Tochigi, 321-0293, Japan; 2 Department of Neurology, Dokkyo Medical University Koshigaya Hospital, 2-1-50 Minami-Koshigaya, Koshigaya, Saitama, 343-8555, Japan

**Keywords:** Parkinson’s disease, Rapid eye movement sleep behavior disorder, Cognition, Quality of life, Nocturnal problems

## Abstract

**Background:**

Increasing evidence provides a clear association between rapid eye movement sleep behavior disorders (RBD) and Parkinson’s disease (PD), but the clinical features that determine the co-morbidity of RBD and PD are not yet fully understood.

**Methods:**

We evaluated the characteristics of nocturnal disturbances and other motor and non-motor features related to RBD in patients with PD and the impact of RBD on their quality of life. Probable RBD (pRBD) was evaluated using the Japanese version of the RBD screening questionnaire (RBDSQ-J).

**Results:**

A significantly higher frequency of pRBD was observed in PD patients than in the controls (RBDSQ-J ≥ 5 or ≥ 6: 29.0% vs. 8.6%; 17.2% vs. 2.2%, respectively). After excluding restless legs syndrome and snorers in the PD patients, the pRBD group (RBDSQ-J≥5) showed higher scores compared with the non-pRBD group on the Parkinson’s disease sleep scale-2 (PDSS-2) total and three-domain scores. Early morning dystonia was more frequent in the pRBD group. The Parkinson’s Disease Questionnaire (PDQ-39) domain scores for cognition and emotional well-being were higher in the patients with pRBD than in the patients without pRBD. There were no differences between these two groups with respect to the clinical subtype, disease severity or motor function. When using a cut-off of RBDSQ-J = 6, a similar trend was observed for the PDSS-2 and PDQ-39 scores. Patients with PD and pRBD had frequent sleep onset insomnia, distressing dreams and hallucinations. The stepwise linear regression analysis showed that the PDSS-2 domain “motor symptoms at night”, particularly the PDSS sub-item 6 “distressing dreams”, was the only predictor of RBDSQ-J in PD.

**Conclusion:**

Our results indicate a significant impact of RBD co-morbidity on night-time disturbances and quality of life in PD, particularly on cognition and emotional well-being. RBDSQ may be a useful tool for not only screening RBD in PD patients but also predicting diffuse and complex clinical PD phenotypes associated with RBD, cognitive impairment and hallucinations.

## Background

Rapid eye movement (REM) sleep behavior disorder (RBD) is characterized by the loss of normal muscle atonia during REM sleep and dream-enacting behaviors during REM sleep, and it often leads to injury of the patient or the bed partner [1]. RBD has been reported to predominantly affect males over the age of 50, and it correlates with neurodegenerative disease, particularly alpha synucleinopathies, such as Parkinson’s disease (PD), multiple system atrophy and dementia with Lewy bodies [2]. Schenck and colleagues have reported that 3.7 ± 1.4 years after an initial diagnosis of idiopathic RBD, 38% of patients developed a parkinsonian syndrome [3]. In a recent prospective study of idiopathic RBD patients, the estimated 5-year risk of neurodegenerative disease was 17.7%, the 10-year risk was 40.6% and the 12-year risk was 52.4% [4].

The frequency of RBD is reported to be up to 60% of PD patients [5]. PD patients with RBD have been reported to manifest a characteristic PD subtype that comprises an akinetic-rigid (non-tremor) phenotype with an increased frequency of falls, a poor response to dopaminergic medications, autonomic dysfunction and impaired color vision [6-9]. The Braak staging system for PD [10], which is based on the temporal sequence of alpha synuclein pathology, shows that pathologic changes begin in the medulla and olfactory bulb (stage 1) and ascend to the rostral regions (stage 2), including the sublaterodorsal nucleus, magnocellular reticular formation and peri-locus coeruleus structures, which are associated with RBD [11]; the changes then reach the midbrain (stage 3) and eventually the cortical structures (stages 4 and 5). This staging may help explain how RBD can precede parkinsonism and cognitive decline in PD; however, whether RBD and PD co-morbidity results in more severe or extensive brain involvement than PD alone remains unknown, and whether differences exist between these two groups (RBD and non-RBD groups) in disease severity and motor score is still controversial [6,9,12-16]. Moreover, despite a number of studies that have evaluated daytime motor and non-motor features of RBD in PD, the characteristics of RBD-mediated nocturnal problems remain poorly studied in PD patients.

The current diagnostic criteria for RBD require polysomnography (PSG), which is used to demonstrate REM sleep without atonia [17]. However, given the cost and facilities required for PSG, questionnaires that adequately screen for “probable RBD” are useful for clinical studies [18]. We evaluated nocturnal disturbances and motor and non-motor symptoms associated with the presence of pRBD in PD patients and the impact of RBD on the quality of life (QOL). We used the self-administered Parkinson’s disease sleep scale (PDSS)-2, which is a specific questionnaire for PD-related sleep disturbances [19], and the Japanese version of the RBD screening questionnaire, which is a validated screening tool for RBD (RBDSQ-J) [20,21].

## Methods

### Design and setting

This study is a cross-sectional, case-controlled study conducted at the Department of Neurology, Dokkyo Medical University between January 2011 and May 2011.

### Subjects

The study sample consisted of a consecutive series of 93 PD patients (69.6 ± 8.9 years; 50 men and 43 women) who were recruited from the validation study of the Japanese version of the PDSS-2 [22]. Control subjects with no neurological or psychiatric diseases (93 age- and gender-matched subjects; 69.5 ± 10.2 years; 47 men and 46 women) were recruited from the medical staff and the staff’s friends and family. A diagnosis of PD was established using the UK Parkinson’s Disease Society Brain Bank clinical diagnostic criteria [23]. Patients with secondary parkinsonism due to medications or trauma were excluded. Vascular parkinsonism or atypical Parkinson syndrome, such as multiple system atrophy, progressive supranuclear palsy, corticobasal degeneration or dementia with Lewy bodies, were diagnosed using brain imaging and clinical examinations, and patients with these diagnoses were excluded. Individuals with dementia were diagnosed using DSM-IV criteria and were excluded [24]. Bedridden patients or patients who were unable to answer the questionnaire (even with the help of their caregiver or family) were also excluded from this study.

### Clinical assessment

The disease severity was rated using the Hoehn and Yahr (HY) staging criteria [25]. Parts III and IV of the Unified Parkinson’s Disease Rating Scale (UPDRS) were used to evaluate the motor scores and complications of treatment, respectively [26]. The UPDRS III score for each cardinal motor feature was also evaluated as a proportion of the total UPDRS part III score and included the following: tremors, the total score for items 20 and 21 (seven items); rigidity, item 22 (five items); bradykinesia, items 23–26 and 31 (nine items); gait/postural stability, items 27–30 (four items) and bulbar abnormalities, items 18 and 19 (two items) [6].

### Clinical subtype

Motor phenotypes with tremor and non-tremor symptoms were compared as previously described [27]. The tremor score was derived from the sum of UPDRS items 20 (tremor at rest) and 21 (action or postural tremor of hands) divided by 7 (the number of single sub-items). The non-tremor score was derived from the sum of UPDRS items 18 (speech), 19 (facial expression), 22 (rigidity), 27 (arising from chair), 28 (posture), 29 (gait), 30 (postural stability) and 31 (body bradykinesia and hypokinesia) divided by 12 (the number of single sub-items). The tremor/non-tremor ratio was then calculated.

### Axial-limb ratio

To compare the regional components of parkinsonism, the UPDRS part III score was subdivided into axial and limb divisions [6]. Axial signs were defined as UPDRS items 18, 19, 22 and 27–30, and limb signs were defined as items 20–26. The axial/limb ratio was calculated as the summed axial scores/summed limb scores.

### Questionnaires

Family members or care givers were allowed to help the PD patients fill out their questionnaires. The Japanese version of the RBD screening questionnaire (RBDSQ-J) was used to determine probable RBD (pRBD) in this study [28]. The RBDSQ is a recently developed self-rating instrument, comprising the following 10 items on the most prominent clinical features of RBD [21]: items 1 to 4, the frequency and content of dreams and their relationship to nocturnal movements and behavior; item 5, self-injuries and injuries to the bed partner; item 6, four sub-items that more specifically assess nocturnal motor behavior, e.g., questions on nocturnal vocalization (6.1), sudden-limb movements (6.2), complex movements (6.3) or bedding items that fall down (6.4); items 7 and 8, nocturnal awakenings; item 9, general disturbances of sleep; and item 10, the presence of any neurological disorder. Each item may be answered using either “yes” or “no”. The maximum total score of the RBDSQ is 13 points. An RBDSQ score cut-off of 5.0 has been considered useful for differentiating idiopathic RBD patients from controls [21,28]. Nomura et al. [29] recently reported that applying a cut-off of 6 in patients with PD and RBD showed better discrimination from those PD patients without RBD. However, PD patients with RBD frequently exhibit a less severe form of abnormal behavior during REM sleep compared with patients with idiopathic RBD [5,30]. We therefore grouped patients using an RBDSQ-J cut-off of 5 or 6. Next, we excluded patients with restless legs syndrome and snorers from the patients with pRBD and those without pRBD. We then compared the clinical profiles and demographic data of the patients with both settings.

The QOL was evaluated using the Parkinson’s Disease Questionnaire (PDQ-39) [31]. The PDQ-39 consists of 39 items that address 8 dimensions: mobility (10 items), activities of daily living (ADL, 6 items), emotional well-being (6 items), stigma (4 items), social support (3 items), cognition (4 items), communication (3 items) and bodily discomfort (3 items). The scores for each dimension and the summary index (SI), which is the sum of the eight dimension scores, were calculated on a scale from 0 (perfect health as assessed by this measure) to 100 (worst health as assessed by this measure). The Beck Depression Inventory (BDI)-II was used to evaluate depressive symptoms [32].

The Japanese version of the PDSS-2 was used to evaluate nocturnal disturbances [22]. The PDSS-2 consists of 15 individual items that measure nocturnal disturbance [19]. The scores for each item assess the frequency of symptoms and range from 0 (never) to 4 (very often). The total score ranges from 0 (symptom-free) to 60 (highly symptomatic). The PDSS-2 was further divided into three domain scores by summing groups of 5 individual item scores (for a maximum score of 20):“disturbed sleep” (items 1–3, 8 and 14); “motor symptoms at night” (items 4–6, 12 and 13); and “PD symptoms at night” (items 7, 9–11 and 15). Snoring was defined as a snoring frequency of ≥ 2 days per week (scores of 2 or higher for PDSS-2 item 15 (breathing problems)). The Pittsburgh sleep quality index (PSQI) was used to evaluate the quality of sleep [33]. The following seven component scores were also estimated (range of subscale scores, 0–3): C1, sleep quality; C2, sleep latency; C3, sleep duration; C4, habitual sleep efficiency; C5, sleep disturbances; C6, use of sleeping medications; and C7, daytime dysfunction. Daytime sleepiness was measured using the Japanese version of the Epworth sleepiness scale (ESS) [34]. Restless legs syndrome (RLS) was diagnosed using four essential criteria for RLS, as previously described by the International Restless Legs Syndrome Study Group [35]. The Parkinson fatigue scale (PFS) was used to assess PD-mediated fatigue [36,37]. The levodopa equivalent dose (LED) was calculated based on findings reported in a systematic review on LED [38].

This study was performed in accordance with the Declaration of Helsinki and was approved by the institutional review boards of Dokkyo Medical University. Written, informed consent was obtained from all subjects enrolled in this study.

### Statistical analysis

The Mann–Whitney *U* test and unpaired *t* test were used, as appropriate, to compare continuous variables, and the Chi-Squared and Fisher’s exact tests were used to compare frequencies between groups. Spearman rank correlation coefficients were used to assess the correlation between RBDSQ-J and other variables. Stepwise linear regression analyses, which included age, gender, disease duration, UPDRS III, the presence of motor complications, PSQI, ESS, BDI-II, the presence of RLS, PFS, LED and PDSS-2 domain scores, were performed to analyze the determinants of the RBDSQ-J score. Significant differences were two-tailed, with *P* < 0.05. A commercially available software package (IBM SPSS Statistics 19.0, Tokyo, Japan) was used for the statistical analyses.

## Results

The mean disease duration in the PD patients was 6.8 ± 6.1 years, and the mean HY stage was 2.6 ± 0.8. The UPDRS III and UPDRS IV scores were 23.0 ± 13.9 and 1.8 ± 2.3, respectively. Motor complications were observed in 35 patients (37.6%). Wearing-off phenomenon, early morning dystonia and dyskinesia were present in 31 (33.3%), 7 (7.5%) and 18 (19.4%) patients, respectively. Eight patients were drug naïve, 11 and 16 patients were undergoing dopamine agonist and levodopa monotherapies, respectively, and 58 patients were being treated with a combination of levodopa and a dopamine agonist. The clinical profiles of the PD patients and controls are shown in Table 1. The PD patients showed a significantly higher RBDSQ-J score than the controls. The PD patients also exhibited a significant increase in the frequency of pRBD compared with the controls (RBDSQ-J ≥ 5 or ≥ 6: 29.0% vs. 8.6%; 17.2% vs. 2.2%, respectively). The PSQI global score showed a tendency to be higher in the PD patients than in the controls. The ESS, BDI-II and PFS scores were significantly different in the PD patients compared with the controls, and the mean PDSS-2 total and domain scores in the PD patients were higher than in the controls.

**Table 1:** The clinical characteristics of the controls and PD patients

	**PD**	**Controls**	**p value**
N (M/F)	93, (50/43)	93, (47/46)	0.66
Age (yrs)	69.7 ± 8.9	69.6 ± 10.2	0.94
Body mass index (kg/m^2^)	22.3 ± 2.8	22.9 ± 3.1	0.18
* RLS, n (%)	5 (5.5)	2 (2.2)	0.44
Snoring (at least 2 days/week), n (%)	13 (14.0)	1 (1.1)	**0.0010**
RBDSQ-J ≥ 5, n (%)	27 (29.0)	8 (8.6)	**<0.001**
RBDSQ-J ≥ 6, n (%)	16 (17.2)	2 (2.2)	**<0.001**
RBDSQ-J	3.3 ± 2.5	1.7 ± 1.7	**<0.001**
PSQI global score	5.4 ± 3.7	4.5 ± 3.2	0.068
PSQI component score			
Sleep quality	1.0 ± 0.7	0.8 ± 0.6	**0.024**
Sleep latency	0.8 ± 1.0	1.0 ± 1.0	0.40
Sleep duration	0.8 ± 0.9	0.8 ± 0.9	0.62
Habitual sleep efficiency	0.5 ± 0.9	0.5 ± 0.8	0.67
Sleep disturbances	0.9 ± 0.6	0.8 ± 0.6	0.47
Use of sleeping medication	0.7 ± 1.2	0.6 ± 1.1	0.48
Daytime dysfunction	0.7 ± 0.9	0.2 ± 0.4	**<0.001**
ESS	6.6 ± 4.5	4.7 ± 3.0	**<0.001**
PDSS-2 total score	15.0 ± 9.7	9.1 ± 6.6	**<0.001**
PDSS-2 domain score			
Disturbed sleep	7.6 ± 3.5	5.6 ± 3.4	**<0.001**
Motor symptoms at night	3.9 ± 4.1	1.8 ± 2.5	**<0.001**
PD symptoms at night	3.5 ± 3.6	1.6 ± 2.1	**<0.001**
PDQ-39 summary index	29.1 ± 21.7	NA	
PDQ-39 domain score			
Mobility	40.7 ± 33.9	NA	
ADL	33.0 ± 29.4	NA	
Emotional well-being	29.2 ± 25.8	NA	
Stigma	22.1 ± 22.4	NA	
Social support	12.0 ± 20.9	NA	
Cognition	23.6 ± 21.2	NA	
Communication	17.3 ± 20.8	NA	
Bodily discomfort	25.1 ± 24.8	NA	
BDI-II	13.3 ± 9.3	8.5 ± 6.8	**<0.001**
PFS	2.8 ± 1.1	1.7 ± 0.8	**<0.001**
Levodopa (mg/day)	304.8 ± 135.5	NA	
LED (mg/day)	385.1 ± 315.0	NA	

PD, Parkinson’s disease; RLS, restless legs syndrome; RBDSQ-J, Japanese version of the REM sleep behavior disorder screening questionnaire; PSQI, Pittsburgh Sleep Quality Index; ESS, Epworth Sleepiness Scale; PDSS-2, Parkinson’s Disease Sleep Scale-2; PDQ-39, Parkinson’s Disease Questionnaire; BDI-II, Beck Depression Inventory-II; PFS, Parkinson Fatigue Scale; LED, Levodopa equivalent dose; NA, not applicable. *Data are missing for 4 controls and 2 PD patients.

The data are presented as the n (%) or the means ± SD. Statistically significant values (P < 0.05) are shown in bold.

Table 2 shows the clinical characteristics of PD patients with pRBD and those without pRBD after the exclusion of RLS and snorers (defined using RBDSQ-J ≥ 5). The pRBD group showed a higher rate of early morning dystonia and higher scores of UPDRS IV and PDSS-2 total scores than the non-pRBD group. The PDQ-39 domain scores for cognition and emotional well-being were higher in the patients with pRBD than in those without. In patients with and without pRBD, the use of entacapone, selegiline, trihexyphenidyl, atypical neuroleptics, antiepileptics and antidepressants, including selective serotonin reuptake inhibitors, tricyclics and tetracyclics, did not differ.

**Table 2:** The clinical parameters of PD patients with and without pRBD

	**pRBD (RBDSQ-J ≥ 5)**	**Non-pRBD (RBDSQ-J < 5)**	**p value**
n; (M/F)	18; (9/9)	59; (31/28)	0.85
Age (yrs)	71.4 ± 5.0	68.3 ± 9.8	0.34
Body mass index (kg/m^2^)	22.8 ± 2.4	22.3 ± 2.8	0.72
Disease duration (yrs)	7.8 ± 5.5	6.4 ± 6.1	0.20
Hoehn and Yahr stage	2.7 ± 0.9	2.5 ± 0.8	0.49
UPDRS III	24.5 ± 12.0	19.9 ± 13.0	0.16
UPDRS IV	2.4 ± 2.4	1.6 ± 2.3	**0.037**
Motor complications, n (%)	8 (44.4)	19 (32.2)	0.34
Wearing-off, n (%)	5 (27.8)	18 (30.5)	1.0
Early morning dystonia, n (%)	4 (22.2)	0 (0.0)	**0.0023**
Dyskinesia, n (%)	5 (27.8)	10 (16.9)	0.32
PSQI global score	5.6 ± 4.0	5.1 ± 3.4	0.76
PSQI component score			
Sleep quality	1.1 ± 0.8	0.9 ± 0.6	0.38
Sleep latency	0.9 ± 1.1	0.8 ± 0.9	0.54
Sleep duration	0.5 ± 0.8	0.9 ± 0.9	0.05
Habitual sleep efficiency	0.4 ± 0.7	0.5 ± 0.9	0.87
Sleep disturbances	0.8 ± 0.6	0.8 ± 0.6	0.83
Use of sleeping medication	0.9 ± 1.3	0.6 ± 1.1	0.14
Daytime dysfunction	0.8 ± 1.0	0.6 ± 0.8	0.39
ESS	7.3 ± 4.7	6.2 ± 4.5	0.34
PDSS-2 total score	16.1 ± 7.4	12.1 ± 7.9	**0.044**
PDQ-39 summary index	31.2 ± 24.4	23.8 ± 19.2	0.25
PDQ-39 domain score			
Mobility	38.2 ± 35.8	33.9 ± 31.3	0.69
ADL	36.1 ± 33.4	27.4 ± 27.2	0.39
Emotional well-being	37.0 ± 27.2	22.4 ± 21.9	**0.029**
Stigma	22.2 ± 19.1	19.1 ± 21.9	0.22
Social support	12.0 ± 22.4	8.8 ± 16.6	0.44
Cognition	31.6 ± 21.1	17.5 ± 18.9	**0.0038**
Communication	19.4 ± 22.0	13.7 ± 18.2	0.27
Bodily discomfort	28.2 ± 25.4	21.5 ± 24.0	0.33
BDI-II	14.2 ± 9.1	11.6 ± 9.2	0.22
PFS	2.7 ± 1.1	2.7 ± 1.1	0.78
Levodopa (mg/day)	270.8 ± 135.6	315.7 ± 154.0	0.44
LED (mg/day)	312.9 ± 234.7	372.5 ± 338.0	0.87

PD, Parkinson’s disease; pRBD, probable REM sleep behavior disorder; UPDRS, Unified Parkinson’s Disease Rating Scale; RLS, restless legs syndrome; RBDSQ-J, Japanese version of the RBD screening questionnaire; PSQI, Pittsburgh Sleep Quality Index; ESS, Epworth Sleepiness Scale; PDSS-2, Parkinson’s Disease Sleep Scale-2; PDQ-39, Parkinson's Disease Questionnaire; BDI-II, Beck Depression Inventory-II; PFS, Parkinson Fatigue Scale; LED, Levodopa equivalent dose. The data are presented as the n (%) or the means ± SD. Statistically significant values (P < 0.05) are shown in bold. Patients with RLS and snorers were excluded.

With respect to the PDSS-2 domain scores and sub-items, patients with both PD and pRBD showed frequent sleep onset insomnia (item 2), distressing dreams (item 6) and distressing hallucinations (item 7) (Table 3). There were no differences in the clinical subtypes, such as the tremor/non-tremor ratio, axial/limb ratio and the UPDRS III proportional scores, although the tremor score tended to be higher in the pRBD group (Table 4).

**Table 3:** PDSS-2 domain scores and sub-items in PD patients with and without pRBD

**PDSS-2**	**Item**	**pRBD (RBDSQ-J ≥ 5)**	**Non-pRBD (RBDSQ-J < 5)**	**p value**
**Disturbed sleep**		8.3 ± 3.0	6.7 ± 3.3	0.066
Poor sleep quality	1	1.3 ± 1.4	1.0 ± 1.3	0.35
Difficulty falling asleep	2	1.7 ± 1.5	0.8 ± 1.0	**0.013**
Difficulty staying asleep	3	2.1 ± 1.3	1.8 ± 1.3	0.35
Tired/sleepy in the morning	14	0.9 ± 1.1	0.7 ± 0.9	0.62
Get up to pass urine	8	2.3 ± 1.2	2.4 ± 1.4	0.69
**Motor symptoms at night**		4.7 ± 3.6	2.8 ± 3.3	**0.036**
Restlessness of arms or legs	4	1.2 ± 1.1	0.8 ± 1.1	0.083
Urge to move arms or legs	5	0.8 ± 1.0	0.4 ± 0.8	0.084
Distressing dreams	6	1.3 ± 1.1	0.4 ± 0.8	**<0.001**
Painful posturing in morning	12	0.3 ± 0.7	0.4 ± 1.0	0.86
Tremor on waking	13	0.9 ± 1.4	0.7 ± 1.1	0.84
**PD symptoms at night**		3.1 ± 2.9	2.5 ± 3.0	0.31
Distressing hallucinations	7	0.6 ± 0.9	0.2 ± 0.7	**0.0074**
Uncomfortable and immobile	9	0.8 ± 1.0	0.7 ± 0.9	0.58
Pain in arms or legs	10	0.6 ± 0.8	0.7 ± 1.0	0.88
Muscle cramps in arms or legs	11	0.7 ± 0.8	0.6 ± 1.0	0.47
Breathing problems/snoring	15	0.4 ± 0.5	0.3 ± 0.5	0.60

PD, Parkinson’s disease; pRBD; probable REM sleep behavior disorder; PDSS-2, Parkinson’s Disease Sleep Scale-2. The data are presented as the n (%) or the means ± SD. Statistically significant values (P < 0.05) are shown in bold. Patients with RLS and snorers were excluded.

**Table 4:** Comparison of motor features in PD patients with and without pRBD

	**pRBD (RBDSQ-J ≥ 5)**	**Non-pRBD (RBDSQ-J < 5)**	**p value**
Tremor score	0.4 ± 0.4	0.2 ± 0.3	0.062
Non-tremor score	1.0 ± 0.5	1.0 ± 0.6	0.73
Tremor/non-tremor score	0.5 ± 0.4	0.4 ± 0.6	0.079
Bulbar (% of total UPDRS III)	10.3 ± 6.3	10.8 ± 10.2	0.66
Tremor (% of total UPDRS III)	12.3 ± 9.4	9.6 ± 13.8	0.087
Rigidity (% of total UPDRS III)	22.8 ± 12.5	21.6 ± 14.8	0.54
Bradykinesia (% of total UPDRS III)	40.4 ± 11.4	40.2 ± 15.5	0.71
Gait posture abnormality (% of total UPDRS III)	14.2 ± 9.9	17.8 ± 13.3	0.35
Axial score	11.0 ± 5.6	10.3 ± 6.2	0.65
Limb score	16.2 ± 7.7	13.8 ± 9.7	0.19
Axial/limb ratio	0.7 ± 0.2	0.9 ± 0.5	0.20

PD, Parkinson’s disease; pRBD; probable REM sleep behavior disorder; UPDRS, Unified Parkinson’s Disease Rating Scale. Patients with RLS and snorers were excluded.

When using a cut-off of RBDSQ-J ≥ 6, the frequency of pRBD was 13.0% (after the exclusion of RLS and snorers; 10/77). No significant differences between these groups were found with respect to age, body-mass index, disease duration, HY stage, UPDRS III or IV, PSQI, ESS, BDI-II, PFS or LED. The PDSS total score tended be higher (17.4 ± 8.4 vs. 12.4 ± 7.7, *p* = 0.073), and early morning dystonia (20.0% vs. 0.0%, *p* = 0.073) tended be more frequent in the pRBD group than the non-pRBD group. The PDSS-2 domain “motor symptoms at night” (5.4 ± 3.9 vs. 2.9 ± 3.3, *p* = 0.039), item 6 (1.6 ± 1.3 vs. 0.5 ± 0.9, *p* < 0.001) and item 7 (0.8 ± 1.0 vs. 0.2 ± 0.7, *p* = 0.0034) were higher in the pRBD group than in the non-pRBD group. Additionally, muscle cramps in the arms or legs (item 11) (1.1 ± 0.9 vs. 0.6 ± 1.0, *p* = 0.043) were more frequent in the pRBD group. The PDQ-39 emotional well-being, cognition, stigma and communication domains were significantly impaired in the pRBD group compared with the non-pRBD group. There were no differences in the clinical subtypes between these two groups.

Among the 77 PD patients without RLS and who were not snorers, the RBDSQ-J score was significantly correlated with the UPDRS IV (*r*_*s*_ = 0.28, *p* = 0.013), PDSS-2 total (*r*_*s*_ = 0.35, *p* = 0.0016) and all-domains scores (“disturbed sleep”, *r*_*s*_ = 0.33, *p* = 0.0033; “motor symptoms at night”, *r*_*s*_ = 0.30, *p* = 0.0072 and “PD symptoms at night”, *r*_*s*_ = 0.24, *p* = 0.035), PDQ-39 emotional well-being (*r*_*s*_ = 0.29, *p* = 0.011), cognition (*r*_*s*_ = 0.46, *p* < 0.001) and bodily discomfort (*r*_*s*_ = 0.27, *p* = 0.022) and BDI-II score (*r*_*s*_ = 0.24, *p* = 0.035). There were significant correlations with most of the PDSS sub-items except for items 1, 3, 8 and 13. There were no correlations between the RBDSQ-J score and the UPDRS III score, disease duration, HY stage, PSQI, ESS, PFS, LED or any clinical subtypes, such as the tremor/non-tremor score and axial/limb ratio.

Stepwise linear regression analyses, which included age, gender, disease duration, UPDRS III and IV, PSQI, ESS, BDI-II, the presence of RLS, PFS, LED and PDSS-2 domain scores, revealed that the PDSS-2 domain “motor symptoms at night” was the only predictor for RBDSQ-J in PD (R^2^ = 0.078, *p* = 0.016). Next, to determine which sub-item of the PDSS-2 domain “motor symptoms at night” contributed to RBDSQ-J, we performed a second stepwise linear regression analysis including age, gender, disease duration, UPDRS III, presence of motor complications, BDI-II, PSQIG, ESS, PDSS sub-items of motor symptoms at night (items 4, 5, 6, 12 and 13), PFS and LED. The results showed that PDSS-2 item 6 “distressing dreams” was the significant contributing factor to RBDSQ-J (R^2^ = 0.078, *p* = 0.016).

## Discussion

In this questionnaire-based study, we found a significantly increased frequency of pRBD in PD patients compared with a control population. The frequency of RBD in our study is comparable with that observed in previous studies. In 469 non-demented Japanese PD patients, the pRBD comorbidity was noted in 31.6% of the individuals (defined using RBDSQ-J ≥ 5) [39].

In our study, a cut-off score of 5 or 6 on the RBDSQ-J revealed similar clinical characteristics in the PD patients. The original study of the RBDSQ showed that a cut-off of 5 was useful for differentiating idiopathic RBD patients from healthy subjects, showing a sensitivity of 96% and a specificity of 56% [21]. Similarly, in our previous validation study of the RBDSQ, an RBDSQ-J cut-off of 5 was useful for differentiating Japanese idiopathic RBD patients from healthy subjects, with a sensitivity of 88.5% and specificity of 96.9% [28]. Recently, Nomura et al. [29] reported upon the usefulness of the RBDSQ-J in Japanese PD patients. In that study, a cut-off point of 6 yielded a sensitivity of 84.2% and a specificity of 96.2%. This finding is in contrast with that of a study assessing the sensitivity of clinical interviews (minimal diagnostic criteria of ICSD, revised) for diagnosing PSG-confirmed RBD in PD patients and controls, which revealed that in PD patients, a low sensitivity (33%) and high specificity (90%) was observed whereas in controls, an excellent sensitivity and specificity were noted (sensitivity, 100%; specificity, 99.6%). The authors suggested that these findings were due to common observations of the mild form of RBD and unawareness of sleep problems and other sleep-related disturbances in patients with PD [40]. The RBDSQ is, therefore, thought to have superior sensitivity for detecting RBD, even in PD patients. In the original version of the RBDSQ, comorbid neurologic diseases such as PD were included in item 10. Patients with PD and RBD frequently exhibit a less severe form of abnormal behavior during REM sleep compared with individuals with idiopathic RBD [5,30]. Thus, we believe that applying a cut-off score of 5, which can increase the sensitivity for detecting pRBD, may be suitable for PD patients, and thereafter, PSG may be used upon suspicion of RBD and when the patient is considered suitable for PSG examination.

There were no significant differences in the gender ratio, disease duration, disease severity, UPDRS III scores or PFS scores between the pRBD and non-pRBD groups. Furthermore, the axial-limb ratio was similar between the groups, a finding similar to that of Postuma et al. [6]; however, they reported that patients with PD who had a symmetric onset showed increased RBD comorbidity and an increased percentage of tonic REM sleep compared with those showing an asymmetric onset [41]. Several, but not all [6,9,15], studies [13,14,16,42] have reported that patients with RBD and PD manifest with non-tremor-dominant type PD rather than tremor-dominant type PD. This finding may imply a more extensive brain involvement in those PD patients with RBD than in those without because in non-tremor-dominant type PD, patients show a more rapid motor deterioration whereas tremor-dominant cases show a benign disease course [43]. Moreover, compared with the tremor-dominant PD type, the akinetic-rigid type (non-tremor-dominant type) showed severe neuronal loss in the medial and lateral substantia nigra and locus coeruleus [44]. In contrast, we found that disease duration, motor scores and levodopa treatment did not differ between the two PD groups, and we did not find a specific motor subtype that correlated with pRBD, although there was a trend towards a higher tremor score in the pRBD group compared with the non-pRBD group. Similarly, a study of 457 PD patients with sleep disturbances did not find a characteristic clinical subtype for PD with RBD but did report a higher disease severity and longer disease duration in PD patients with RBD than those without [13]. Because most of our patients were medicated, we cannot exclude that a tremor-dominant type may transition into a non-tremor-dominant type over time and with increased age [45]. In early PD (disease duration of up to 5 years and HY stage of between 1 and 2.5), the RBD co-morbidity (confirmed by clinical history during the preceding 6 months and a cut-off score > 4 on the RBDSQ) was high (55%); however, no differences in the clinical subtype and disease severity have been reported recently [42].

Our study demonstrated that the scores for the PDQ-39 cognition and emotional well-being dimensions were higher in patients with pRBD than in those without pRBD. Patients with idiopathic RBD have no cognitive or motor complaints, but they frequently develop characteristic motor and cognitive symptoms, such as impairment of visuospatial abilities and deficits in verbal memory, attention and executive function, which are also observed in patients with PD, PD with dementia, MCI and DLB [46]. Several risk factors associated with dementia in PD, such as the akinetic-rigid type, hallucinations, longer disease duration and male gender, correlate with RBD in PD [46]. In our study, hallucinations also correlated significantly with PD-RBD.

The negative impact of RBD on the QOL has been previously reported [7]. In that report, no changes were found in the PDQ-39 score, but lower QOL scores were noted (these scores were measured using the 36-item short form health survey on emotional and mental functioning) in PD patients with RBD, suggesting that RBD in PD may affect the QOL in a non-disease-specific manner. In contrast, in our study, similar components of the QOL were affected in the PDQ-39, the disease-specific QOL questionnaire for PD. Because the pRBD group showed higher PDSS scores and PDQ-39 cognition dimensions than the group without pRBD, impaired sleep may play a role in cognitive dimensions in this study.

We found that PD patients with pRBD showed significantly higher PDSS-2 scores related to sleep onset insomnia, nocturnal hallucinations and vivid dreams but that the ESS and PSQI scores were similar between the groups. The impairment of REM sleep has been attributed to the occurrence of visual hallucinations and delusions in PD [47]. RBD is a possible predictor for the development of cognitive impairment and visual hallucinations in PD, and RBD, hallucinations and cognitive impairment may progress together with motor impairment [48,49]. Because LED and disease severity were not different between the pRBD and non-pRBD groups, these nocturnal dysfunctions may be related to impairments in non-dopaminergic systems.

The limitations of our study include the absence of PSG examinations, which may have resulted in an underestimation of the frequency of RBD because the frequency of RBD, diagnosed using clinical history alone, ranges from 15% to 46% whereas the frequency of PSG-confirmed RBD varies from 46% to 58% in PD patients [5]. In addition, the absence of any significant difference in several clinical characteristics and sleep parameters in this study between the PD patients and controls and between the pRBD and non-pRBD groups may be due to insufficient statistical power.

## Conclusions

In conclusion, our results suggest a significant impact of RBD co-morbidity on night time disturbances (as assessed by PDSS-2) and the QOL, especially cognition and emotional well-being, in PD. The RBDSQ, which addresses the most prominent clinical features of RBD, may be a useful tool for not only screening RBD in PD but also predicting diffuse and complex clinical PD phenotypes associated with RBD, cognitive impairment and hallucination.

## Competing interests

The authors declare that they have no competing interests.

## Authors’ contributions

KS made substantial contributions to the conception and design, acquisition of the data, and analysis and interpretation of the data and was involved in drafting the manuscript; TM and MM made substantial contribution to the conception and design, acquisition of the data, and analysis and interpretation of the data and provided critical revisions of the manuscript for important intellectual content. YW, SS, MT, MI, TS, TK, AN, KH and HS made substantial contributions to the acquisition of the data. KH supervised this study. All authors read and approved the final manuscript.

## Pre-publication history

The pre-publication history for this paper can be accessed here:

http://www.biomedcentral.com/1471-2377/13/18/prepub
